# Second-tier strategies in newborn screening – potential and limitations

**DOI:** 10.1515/medgen-2022-2117

**Published:** 2022-05-07

**Authors:** Gwendolyn Gramer, Georg F. Hoffmann

**Affiliations:** University Medical Center Hamburg-Eppendorf, University Children’s Hospital, Martinistraße 52, 20246 Hamburg, Germany; University Hospital Heidelberg, Center for Pediatric and Adolescent Medicine, Division of Neuropediatrics and Metabolic Medicine, Im Neuenheimer Feld 430, 69120 Heidelberg, Germany

**Keywords:** newborn screening, second-tier, prevention, metabolites, genetic screening

## Abstract

Newborn screening (NBS) is a public health measure to identify children with treatable disorders within the first days of life allowing presymptomatic treatment. It is the most successful measure of secondary medical prevention and part of public health programs in many countries worldwide. Application of second-tier strategies in NBS allows for increased specificity and consecutively a higher positive predictive value. Second-tier strategies can include analysis of specific biomarkers for a target disorder or may be based on molecular genetic analyses. Improving the quality of NBS, for example by second-tier strategies, is of utmost importance to maintain the high acceptance of NBS by families – especially as an increasing number of target disorders is being consecutively included into NBS programs.

## Background

Newborn screening (NBS) is a population-based program of secondary prevention. It is performed to allow for early detection of treatable conditions. As this procedure is performed on a large cohort of healthy newborns in order to identify the smaller group of affected individuals in a population, high standards of specificity have to be achieved.

NBS in Germany started in 1964 with screening for phenylketonuria. In the following decades disorders like classical galactosemia, biotinidase deficiency, and the endocrinopathies congenital hypothyroidism and congenital adrenal hyperplasia (CAH) were added [1]. For each of these disorders separate tests for biomarkers or enzyme activities had to be performed in separate punches from the dried blood spot (DBS) specimen. The introduction of electrospray ionization–tandem mass spectrometry (ESI-MS/MS) in the 1990s allowed for a considerable expansion of NBS. This technology allows the analysis of more than 60 metabolites of fat and protein metabolism. Therefore ESI-MS/MS can be used for the early detection of a large number of relatively rare metabolic disorders with a single analytical step. The use of ESI-MS/MS led to a fundamental change in many screening programs [2], [3]. However, every additional target disorder usually leads to an increase of false-positive NBS results (positive NBS in a child not affected by the target disorder).

NBS in Germany currently includes 19 target disorders: 13 metabolic disorders, two endocrinopathies, cystic fibrosis (CF), severe combined immunodeficiencies (SCID), and – since October 2021 – also sickle cell disease and spinal muscular atrophy. The current target disorders of NBS in Germany and screening markers used are listed in Table 1. Other countries like, e. g., the US differentiate between primary target disorders and so-called secondary target disorders [4], which can be detected by NBS but do not necessarily fulfill all classical screening criteria [5]. The recommended Uniform Screening Panel for the US can be accessed in detail on the web site of the U.S. Health Resources & Services Administration (www.hrsa.gov/advisory-committees/heritable-disorders/rusp/index.html).

**Table 1:** Current target disorders of newborn screening in Germany (as of October 2021) and screening markers used.

Disorders		Primary screening markers	Second-/third-tier markers
*Endocrine disorders*	Congenital hypothyroidism	TSH	

	Congenital adrenal hyperplasia	17-OH-progesterone	Steroid profile (used in single German laboratories)
*Metabolic disorders*	Biotinidase deficiency	Biotinidase activity	

	Galactosemia (classical)	GALT activity	Total galactose

	Phenylketonuria/hyperphenylalaninemia (including cofactor deficiencies)	Phenylalanine	
	Tyrosinemia type I	Succinylacetone	

	Maple syrup urine disease	Xle (leucine + isoleucine + alloisoleucine + OH-proline)	Alloisoleucine – in principle available as second-tier test but not routinely used in German laboratories [6]

	Glutaric aciduria type I	Glutarylcarnitine	

	Isovaleric aciduria	Isovalerylcarnitine (C5)	

	Medium-chain acyl-CoA dehydrogenase deficiency	Octanoylcarnitine (C8)	–

	Long-chain 3-OH-acyl-CoA dehydrogenase deficiency	C16OH, C18:1OH	

	Very long-chain acyl-CoA dehydrogenase deficiency	C14:1	

	Carnitine palmitoyltransferase I deficiency	C0, decreased long-chain acylcarnitines	

	Carnitine palmitoyltransferase II deficiency	Long-chain acylcarnitines (C16–C18:2)	

	Carnitine acylcarnitine translocase deficiency	Long-chain acylcarnitines (C16–C18:2)	
*Cystic fibrosis*		IRT	PAP (second tier)
			31 *CFTR* mutations (Germany) or *CFTR* sequencing (second or third tier [7])
*Severe combined immunodeficiencies (SCID)*		TREC (qPCR)	
*Sickle cell disease (SCD)*		HbS	
*Spinal muscular atrophy (SMA)*		SMN1, homozygous exon 7 deletions (qPCR)	

Abbreviations: C_x_ = respective chain length of acylcarnitines; CFTR = cystic fibrosis transmembrane conductance regulator; GALT = galactose-1-phosphate uridyltransferase; TREC = T-cell receptor excision circles; IRT = immunoreactive trypsine; TSH = thyroid-stimulating hormone; HbS = hemoglobin S; PAP = pancreatitis-associated protein; SMN = survival motor neuron; qPCR = quantitative polymerase chain reaction.

## Principles and practice of second-tier strategies

So far NBS for most disorders is based on the measurement of biochemical markers from the DBS specimen (Table 1). In ESI-MS/MS NBS for many disorders, in addition to the primary marker metabolite, also ratios between different metabolites and bioinformatics analysis results can increase sensitivity and specificity [8]. Depending on the grade of pathology and the respective target disorder, out-of-range results in the first NBS sample may result in request for another DBS specimen to repeat the NBS tests, targeted confirmatory testing from additional patient specimens in clinical laboratories, or recommendation for prompt clinical evaluation in a children’s hospital or specialized center for the respective target disorder.

NBS for several disorders using conventional marker metabolites is associated with a relatively low specificity, leading to a high number of false-positive NBS results. This is clearly unwanted in population-based NBS programs as false-positive results lead to unnecessary concern for parents and potentially may cause even longer lasting parent–child dysfunction [9]. For example, NBS for disorders of propionate metabolism leads to a high number of false-positive results when based on elevations of propionylcarnitine (C3) alone [10]. This led to the decision of the German Joint Federal Committee (Gemeinsamer Bundesausschuss [G-BA]) to not include NBS for these disorders in the German NBS panel in the year 2005 when extended NBS using ESI-MS/MS was introduced into routine NBS in Germany [11].

**Figure 1:**
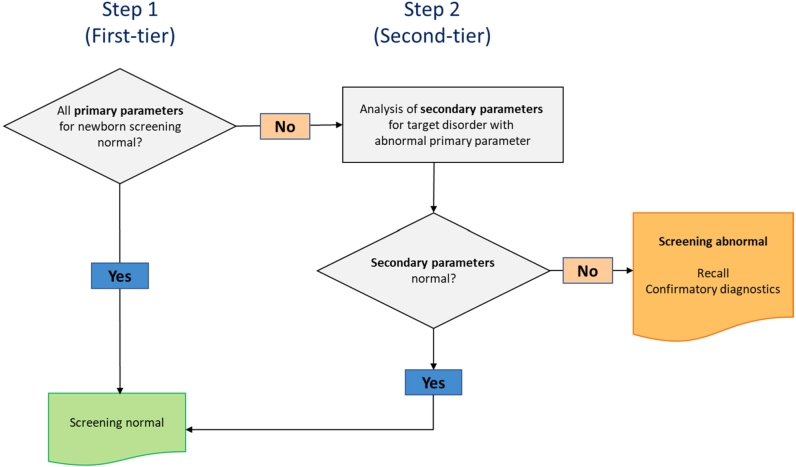
Principle of second-tier strategies in newborn screening (adapted from [12]).

To increase specificity of NBS for disorders without a highly specific primary NBS marker, so-called second-tier strategies have been suggested and developed. In these strategies out-of-range results of the respective primary markers are complemented by measurement of more specific metabolites for the target disorder from the same first NBS specimen (Figure 1). This leads to a much higher specificity and significantly reduces unnecessary requests for repeat DBS collections and the associated concern and anxiety of parents.

On the other hand, NBS by primary marker metabolites based on conventional cut-offs can also be associated with a low sensitivity and therefore may lead to false-negative NBS results (missed cases of truly affected children) for certain disorders. In such cases sensitivity may be increased by using second-tier strategies when cut-offs for non-specific first-tier parameters can be adapted to increase sensitivity due to high specificity of second-tier parameters.

## Second-tier strategies – Biomarkers

### Pilot projects using second-tier strategies

Technical and methodological progress including second-tier strategies and the (assumed) benefit of early detection for affected patients suggest the inclusion of additional target disorders into the German NBS panel.

In NBS for disorders of the propionate metabolism, like methylmalonic acidurias or propionic aciduria, second-tier strategies measuring methylmalonic acid (MMA), 3-OH-propionic acid (3-OH-PA), and methylcitric acid (MCA) from the same first NBS specimen have been applied [13], [14], [15]. In NBS for classical homocystinuria and remethylation disorders, second-tier strategies with measurement of homocysteine (tHcy) from the NBS sample have been successfully evaluated [16], [17], [18].

The application of second-tier strategies for additional NBS target disorders is currently evaluated in Germany in different pilot projects in the NBS laboratories in Munich [19], [20], Hannover, and Heidelberg. These studies evaluate especially technical aspects and feasibility of incorporation of second-tier strategies into the German NBS panel. At the NBS center in Heidelberg, a pilot project called “Newborn screening 2020/2025” is performed since the year 2016, evaluating NBS for 26 additional metabolic disorders and vitamin B_12_ deficiency. In this project second-tier strategies measuring MMA, 3-OH-PA, MCA, and tHcy have been established and evaluated [21], [22]. These second-tier markers can all be measured simultaneously from a single DBS punch. Target disorders screened by second-tier strategies in the context of the pilot project are shown in Table 2. More than 140 patients have already been detected by this study – the largest group were children affected by vitamin B_12_ deficiency. Although not a genetic condition, early detection of vitamin B_12_ deficiency is essential, as it is well treatable but can cause severe neurologic sequelae in infants if untreated. Vitamin B_12_ deficiency in newborns is mostly of maternal origin. Causes in the mother may be unrecognized malabsorption or nutritional deficiency, e. g., due to gastric disease, a vegetarian or vegan diet, or feeding difficulties in pregnancy [23], [24]. Early treatment leads to normal development of affected children [25]. Also, patients with methylmalonic and propionic aciduria or isolated and combined remethylation disorders have been identified by this project, consecutively allowing for early treatment. Some target disorders like classic homocystinuria which seem to be extremely rare in the German population have so far not been detected in the screened population of this project. When finally deciding about the inclusion of additional disorders into the German NBS panel, not only the technical feasibility but also prevalence of the disorder, outcome following early treatment, and cost of the additional screening will play a decisive role.

**Table 2:** Examples of second-tier biomarkers used in newborn screening pilot projects.

Disorder	First-tier	Second-tier
Classical homocystinuria (CBS deficiency)	Methionine/phenylalanine (high)	Total homocysteine (tHcy)
MTHFR deficiency, Cbl-D-Hcy, Cbl E, or Cbl G defect	Methionine (low), methionine/phenylalanine (low)	tHcy
Cbl C, D, F, or J defect, transcobalamin II deficiency	Propionylcarnitine (C3), C3/C2, methionine (low), methionine/phenylalanine (low)	Methylmalonic acid (MMA), methylcitric acid (MCA), tHcy
Methylmalonic acidurias (mut^0^, mut^−^, and Cbl A or Cbl B defect)	Propionylcarnitine (C3), C3/C2	MMA, MCA
Vitamin B_12_ deficiency	Propionylcarnitine (C3), C3/C2, methionine (low), methionine/phenylalanine (low)	MMA, MCA, tHcy
Propionic aciduria	Propionylcarnitine (C3), C3/C2	3-OH-propionic acid, MCA

### NBS for congenital adrenal hyperplasia using second-tier strategies

NBS for CAH is part of NBS panels in many countries, including the program in Germany. NBS for this endocrinopathy is based on levels of 17-OH-progesterone in DBS. Due to the fact that non-specifically elevated levels of this marker metabolite are frequently found in sick or stressed newborns and preterm children, CAH is the target disorder with the highest number of false-positive results in the German NBS panel [26]. Several NBS centers worldwide have demonstrated that the specificity of CAH NBS can be drastically improved by application of second-tier strategies measuring steroid profiles from samples with elevated 17-OH-progesterone [27], [28]. However, false-negative results of CAH NBS have been reported in several cases, irrespective of whether a one- or two-tier screening approach was pursued [29]. The chances for false-negative results for CAH are higher in early collected samples (<48 hours), as is the case in the US, while samples in Germany are taken at 36–72 hours. For potentially decompensating disorders like CAH also the required turnaround time for second-tier tests has to be considered. In highly abnormal first-tier results, a second-tier steroid profile has to be available within 24 hours to allow for timely reporting of results. This also means that batching of samples or transfer to another laboratory for second-tier analysis is not appropriate for these disorders.

**Figure 2:** CF NBS protocol with safety net currently used for the nationwide CF newborn screening program in Germany.

Abbreviations: IRT = immunoreactive trypsin, PAP = pancreatitis-associated protein, CFTR = cystic fibrosis transmembrane conductance regulator; CF = cystic fibrosis; NBS = newborn screening.

## Genetic second-tier and third-tier strategies

### Current use of genetic second-tier and third-tier strategies

In addition, or as an alternative to biochemical second-tier markers, also genetic second-tier or third-tier analyses from the NBS DBS are a promising approach for improved NBS strategies. CF was the first target disorder for which molecular genetic analyses were implemented as part of NBS in different protocols. These are based on the analysis of a limited number of common genetic variants in the *CFTR* gene from the NBS sample. In Germany, a molecular genetic third-tier approach is used for CF NBS [30]. Following this algorithm, an analysis of 31 common genetic variants in the *CFTR* gene is performed in only a small percentage of samples [26] based on out-of-range results for immunoreactive trypsin (IRT) as first-tier and pancreatitis-associated protein (PAP) as second-tier analysis (Figure 2). To prevent patients with rare genetic variants in the *CFTR* gene from being missed by NBS and also in order to minimize the number of molecular genetic analyses, the G-BA decided to implement a “safety net” into the German CF NBS algorithm when CF NBS was initiated in Germany in 2016. This means that in samples with ultrahigh IRT (>99.9 percentile), CF NBS is directly classified as positive without second-tier PAP and third-tier molecular genetic analysis. In the German protocol, already the presence of one genetic variant in the *CFTR* gene in the third-tier analysis will result in a “screen-positive” case requiring further confirmatory work-up. Confirmation of cases as true positive is then primarily based on results of sweat testing – in positive or unequivocal cases complemented by molecular genetic studies or further functional evaluation of CFTR function. Individuals with a mere carrier status but normal sweat test will be classified as false-positives. The G-BA regulated that positive CF NBS results are to be transmitted only as “abnormal” without information whether the sample became positive via the safety net or mutation analysis and on the number of mutations detected. This procedure is explained by the fact that the G-BA set a high value in the attempt not to disclose a mere carrier status of a mutation in the *CFTR* gene in a child of the family.

However, the current protocol for CF NBS in Germany has several pitfalls and limitations while simpler protocols based on only IRT and molecular genetic analyses are in use in several countries worldwide. Due to the safety net approach the German protocol results in a relatively low positive predictive value (PPV) for CF as the diagnosis is confirmed in only one of five screen-positive cases. At the same time several false-negative cases have been reported which could, e. g., be attributed to false-negative PAP results [26]. PPV could be increased by implementation of an IRT/DNA protocol [31]. An evaluation of the current CF NBS protocol has been planned after the first years of CF NBS in Germany but results of this evaluation are still pending.

### Potential and limitation of genetic second-tier and third-tier strategies

There are several additional potential target disorders for NBS for which a second-tier or third-tier molecular genetic screening approach may be promising in combination with biochemical screening for marker metabolites. For example, in the Netherlands a four-tier genetic screening approach for X-linked adrenoleukodystrophy (X-ALD) has been recently included in a gender-specific screening for boys only [32]. NBS for X-ALD has also been established in several US states [33]. However, although molecular genetic testing of *ABCD1* will be provided in the US programs, cases will be reported as presumptive positive already based on the biochemical first- and second-tier tests before sequencing results are available. This approach will therefore also identify other peroxisomal disorders based on the first-tier test.

A second-tier genetic screening could also allow an improved NBS approach for, e. g., neuronal ceroid lipofuscinosis type 2 (CLN2) in addition to enzymatic screening [34]. Also for many other lysosomal disorders second-tier approaches are already in use or a promising future approach to NBS [35].

Norway has introduced second-tier testing by next-generation sequencing in NBS for inborn metabolic disorders and SCID [36]. For other treatable disorders, early detection by NBS would be in principle desirable but is so far not possible due to the lack of specific biomarkers. For disorders like, e. g., cystinosis, glucose transporter-1 (GLUT1) deficiency, or thiamine transporter deficiency, a primary genetic NBS would be a potentially reasonable alternative. We believe that both genetic second-tier approaches and primary genetic screening will play an increasing role in future developments of NBS. However, the potential of primary genetic screening approaches will be limited for disorders with poor genotype–phenotype correlation, very large genes, or a high frequency of variants of uncertain significance [37]. Aspects of primary genetic NBS such as feasibility, and ethical and legal considerations are covered in the following article by Dikow and colleagues.

## Conclusion

NBS is the most successful measure of secondary prevention. It is part of public health programs in many countries worldwide [3]. Application of second-tier strategies in NBS allows for increased specificity and consecutively a higher PPV of NBS. Second-tier strategies can include analysis of specific metabolic markers for a target disorder or may be based on molecular genetic analyses. The attempt for a high quality of NBS, for example by second-tier strategies, is of utmost importance to maintain the high acceptance of NBS by families – especially as an increasing number of target disorders is considered for inclusion into NBS programs.
